# Supplementary material for: Gender differences in adverse childhood experiences, resilience and internet addiction among Tunisian students: Exploring the mediation effect
Source: PLOS Glob Public Health. 2024 Jan 18;4(1):e0002556. doi: 10.1371/journal.pgph.0002556 (PMC10795992; doi:10.1371/journal.pgph.0002556)
Supplement: S2 Data — (DOCX) [file pgph.0002556.s002.docx]

The full list of legends for the supporting information file

| The list of variables | Legends |
| --- | --- |
| Internetaddiction_C1 🡪Internetaddiction_C20 | Internet addiction test items |
| ACE_D1🡪ACE_D2 | Emotional neglect items according to Adverse childhood experiences-International Questionnaire |
| ACE_E1🡪ACE_E7 | Physical neglect items according to Adverse childhood experiences-International Questionnaire |
| ACE_F1🡪ACE_F4 | Household dysfunction items according to Adverse childhood experiences-International Questionnaire |
| ACE_J1🡪ACE_J2 | Emotional abuse items according to Adverse childhood experiences-International Questionnaire |
| ACE_J3🡪ACE_J4 | Physical abuse items according to Adverse childhood experiences-International Questionnaire |
| ACE_J5🡪ACE_J9 | Sexual abuse items according to Adverse childhood experiences-International Questionnaire |
| ACE_H1🡪ACE_H2 | Bullying items according to Adverse childhood experiences-International Questionnaire |
| ACE_I1🡪ACE_I3 | Community Violence items according to Adverse childhood experiences-International Questionnaire |
| ACE_G1🡪ACE_G4 | Collective Violence items according to Adverse childhood experiences-International Questionnaire |
| Resilience_U1🡪Resilience_U29 | The Adolescent Psychological Resilience Scale Items |
| Y_InternetAddiction | A binary variable created from “Score_internetaddiction” (Cyberaddicted, Not Cyberaddicted) |
| Class_physical_abuse | Binary variable created from original physical abuse items |
| Class_emotional_abuse | Binary variable created from original emotional abuse items |
| Class_sexual_abuse | Binary variable created from original sexual abuse items |
| Class_Housholddysfuntion | Binary variable created from original Household dysfunction items |
| Class_emotional_neglect | Binary variable created from original emotional neglect items |
| Class_physical_neglect | Binary variable created from original physical neglect items |
| Class_bulling | Binary variable created from original bullying items |
| Class_community_violence | Binary variable created from original community violence items |
| Class_collective_violence | Binary variable created from original collective violence items |
| XT_ACE | Total score of Adverse Childhood Experiences (ranging from 0 to 9) |
| XI_ACE | Total score of Intrafamilial Adverse Childhood Experiences (ranging from 0 to 6) |
| XE_ACE | Total score of Extrafamilial Adverse Childhood Experiences [ranging from 0 to 3) |
