# Supplementary material for: Gender differences in adverse childhood experiences, resilience and internet addiction among Tunisian students: Exploring the mediation effect
Source: PLOS Glob Public Health. 2024 Jan 18;4(1):e0002556. doi: 10.1371/journal.pgph.0002556 (PMC10795992; doi:10.1371/journal.pgph.0002556)
Supplement: Attachment — Submitted filename: Response to reviewers.docx [file pgph.0002556.s003.docx]

**Response to reviewers**

**Manuscript:**Gender differences in adverse childhood experiences, resilience and internet addiction among Tunisian students: Exploring the mediation effect.

Dear Editor in Chief,

Thank you very much for considering our manuscript to be published in your prestigious journal. Thanks also for the reviewers for their time and valuable comments. Please see below the response to the reviewer comments.

Thank you again for your assistance in this matter

Emna Hariz

**Response to Reviewer 1:**

This manuscript examined the mediating role of resilience in the association between adverse childhood experiences and internet addiction among Tunisian youth. The topic is important and the study has some promise, but there are some major methodological issues that must be considered before any useful conclusions can be made. My specific comments are below.

We are very grateful for your relevant comments and suggestions. They allowed us to improve our manuscript.

-Introduction: This section does not provide justification for considering resilience as a mediator in the association between ACEs and internet addiction. The cited studies support the role of resilience as a moderator (effect modifier) rather than a mediator. By definition, a mediating variable must be on the causal pathway between the exposure and the outcome. Are there studies that support a causal relationship between ACEs and resilience?

It is true that the mediating variable must be on the causal pathway between the exposure and the outcome. Indeed, three studies out of the four mentioned studies supported the role of resilience as a mediator. Concerning the relationship between the ACEs and resilience, two studies were added in the introduction as recommended (Page 4, line 96).

Methods: This section is significantly lacking in details throughout. More information is needed regarding the study sample (how many were recruited, how many consented, response rate)

Yes, it is really important to provide more information regarding the study sample. As you recommended, more details were added in the methods section (Page 6, line 127).

measurement (how were the main variables used in the analysis?)

We understand your concern about how were the main variables used in the analyses. That is why we added more explanations in the measurement tools part (Page 6, line 134).

and statistical methods (see below)
-Statistical methods: No details were provided on the methods used to examine the associations. The authors mention adding the mediator to a model without providing any further details regarding the modeling procedures used.
-How were the numbers in Table 3 obtained?

You are correct, it is crucial to give more information about the statistical methods used to examine the associations. Details were added as requested in the statistical analyses part (Page 7, line 160). Also, we explained more how the numbers in Table 3 were obtained in the Mediation analysis part (Page 7, line 168).

-In the methods section, there was no mention of adjusting for confounding variables. This is a serious flaw in this manuscript that was not even mentioned in the limitations section.

We totally agree. It is important to consider confounding variables such as depression, anxiety and others. This flaw was added in the limitations section as suggested (Page 18, Line 372).

-Results: In table 4, the coefficients include c' which was not mentioned elsewhere.What is this pathway and how was it obtained?

It is true that more precision is needed concerning the pathway c' in the Results section. We overcame the shortage in (Page 12, line 230 and 235) as you suggested. Besides, we would like to draw your attention to the explanation of pathway c' which is provided in the methodology section for your consideration.

-In the interpretation of results, the authors make no mention of the direction of the associations and the implications of these directions on the mediation results.

It is true that mentioning the direction of the associations and the implications of these directions on the mediation results in the interpretation was lacking. Your recommendations were added in the results section, (Page 11, from line 219 to line 224) and (Page 12, from line 230 to line 231).

Discussion: Most of this section is focused on the descriptive results and comparing them to the literature, rather than results that answer the research question (mediation).
-In the discussion, the author's interpretation of the results and their implications is more appropriate for a moderator/effect modifier rather than a mediator. The literature cited in the discussion also supports the role of resilience as a moderator, which would have been a better approach for this analysis than mediation.

It is exact that most of the discussion section is focused on the descriptive results and comparing them rather than results that answer the mediation. That is due to the scarcity of articles performing mediation analysis in the literature. In fact, to the best of our knowledge, we have covered all the studies about the research question. We have pointed out the rarity of studies about the study topic in the introduction section (Page 5, line 110) and the discussion part (Page 17, line 336).

The main limitation, acknowledged by the authors, is the cross-sectional design which does not permit the determination of temporality. This limitation violates the basic assumptions required for mediation analysis, which is another reason why mediation was inappropriate in this case.

We totally agree that the cross-sectional nature of data collection did not provide an understanding of the temporal association between adverse experiences, resilience and internet addiction. However, this limit does not contradict the use of mediation analysis according to literature. Indeed, we found cross-sectional published studies that used the mediation analyses such as references number 14 and 15. Moreover, methodologically, and according to Andrew F. Hayes, the mediation analyses can be conducted using cross-sectional data.

Thank you for your valuable suggestions. We are looking forward to hearing from you and receiving your comments.

Best regards

**Response to Reviewer 2:**

I enjoyed reading about your research and particularly liked the comparative inclusion of data from multiple countries. I would have liked to see the role of ACE and Resilience on overall behavioral addiction mentioned. I assume you find resilience more significant for males in general addiction or other behavioral addictions. Great work.

Thank you for your feedback. We appreciate your suggestion regarding the role of ACE and Resilience in behavioral addiction, particularly in relation to males. This is indeed an interesting avenue for future research.

We are looking forward to hearing from you and receiving your comments.

Best regards

**Response to Reviewer 3:**

Information in the abstract does not match with the title, especially the gender difference. Please modify the introduction and method sections of the abstract.

We agree with you, the introduction and method sections of the abstract were modified as recommended (Page 3, line 52), (Page 3, line 54) and (Page 3, line 61).

Data should be available online as per plos policy.

We understand that, data will be provided.

Please improve the justification of the study.
Add why this study is needed in the context of the country as similar studies are already published.

It is true, the justification of the study should be improved. In deed, we added in the introduction section why this study is needed (Page 5, line 110).

Follow the reporting guidelines STOBE and cover all the sections in the STOBE.
Give description of the population.
Add a section for the questionnaire development.
Add a section for the data collection techniques.
Add a section for the bias.
Add a sections for the dependent variables and independent variables.
Add the exclusion and inclusion criteria.
Report the sampling techniques.
Report the sample size calculation.
Repot the validity and reliability of the tools used in this study.

It is important to follow the reporting guidelines STOBE and cover all the sections in the STOBE. In fact, the exclusion and inclusion criteria and the sampling techniques were added (Page 6, line 124) and (Page 5, line 120), respectively. we already have reported the sample size calculation. Also, the validity and reliability of the tools used in this study were added as recommended (Page 6, line 139) and (Page 7, line 149). However, adding a section for the dependent variables and independent variables is not possible since the mediation model does not involve a traditional dependent or independent variable relationship.

We are looking forward to hearing from you and receiving your comments.

Best regards

**Response to Reviewer 4:**

Qualitative variables were represented by absolute and relative frequencies and quantitative ones were represented by means and standard deviations (SD)” why qualitative variables? Is it a mix method study? If so, how can it be express in frequencies?

Qualitative or categorical variables in our survey are those not numerical such as the gender and the educational level of students. The use of frequencies for qualitative variables is common in many types of research, not just mixed-method studies. It allows for a clear understanding of categorical data patterns. While our study is not mixed-method, employing these measures helps provide comprehensive insights into the data distribution and characteristics within the qualitative variables.

To compare percentages and means, we used the Chi square and the Student tests, respectively” mention why and when this test were done and the purpose of the tests.

The Chi square and the Student tests, were used to compare percentages and means especially in gender comparison (Table 1 and Table 2). We added more precisions as recommended, in the methods section (Page 7, line 158).

The gender is covered male and female that is basically sex. Why not author say it sex difference?

Thank you for your insightful comment. Actually, “Female” is replaced by “Girl” and “Male” is replaced by “Boy” in all the manuscript.

The justification of the sex difference should be mentioned in the justification of the introduction.

Yes, you are right. Justifications were added in the introduction section (Page 4, line 80), (Page 4, line 87) and (Page 5, line 105).

Report the limitation of the study.

The limitation of the study was reported in the discussion section (Page 18, line 361).

Mention what is new in this study in a section.

We estimate that our study is original since the data is lacking in our context (Page 5, line 110).

Give a recommendation. What are the further research in needed, add a section for that.

It is true that recommendations are crucial in every manuscript. Indeed, the discussion section is imbued with recommendations.

We are looking forward to hearing from you and receiving your comments.

Best regards.
