# Supplementary material for: Gender differences in adverse childhood experiences, resilience and internet addiction among Tunisian students: Exploring the mediation effect
Source: PLOS Glob Public Health. 2024 Jan 18;4(1):e0002556. doi: 10.1371/journal.pgph.0002556 (PMC10795992; doi:10.1371/journal.pgph.0002556)
Supplement: Attachment — Submitted filename: Response to reviewers.docx [file pgph.0002556.s004.docx]

**Response to reviewers**

**Manuscript:**Gender differences in adverse childhood experiences, resilience and internet addiction among Tunisian students: Exploring the mediation effect.

Dear Editor in Chief,

Thank you very much for considering our manuscript to be published in your prestigious journal. Thanks also for the reviewers for their time and valuable comments. Please see below the response to the reviewer comments.

Thank you again for your assistance in this matter

Emna Hariz

**Journal requirements:**

We noticed you have some minor occurrence of overlapping text with the following previous publication(s), which needs to be addressed :- <https://www.sciencedirect.com/science/article/pii/S2211335521001145?via%3Dihub> In your revision ensure you cite all your sources (including your own works), and quote or rephrase any duplicated text outside the methods section. Further consideration is dependent on these concerns being addressed.

Thank you for bringing this to our attention. We have carefully reviewed the identified overlapping text with the provided publication. The revised portions are highlighted in fluorescent color for your convenience.

Please provide additional details regarding participant consent. In the ethics statement in the Methods and online submission information, please ensure that you have specified (1) whether consent was informed and (2) what type you obtained (for instance, written or verbal, and if verbal, how it was documented and witnessed). If your study included minors, state whether you obtained consent from parents or guardians. If the need for consent was waived by the ethics committee, please include this information. If you are reporting a retrospective study of medical records or archived samples, please ensure that you have discussed whether all data were fully anonymized before you accessed them and/or whether the IRB or ethics committee waived the requirement for informed consent. If patients provided informed written consent to have data from their medical records used in research, please include this information.

Thank you for your careful and valuable feedback. We appreciate the opportunity to address your concerns regarding participant consent in our study. Details concerning ethics are in the Ethical concerns part (Page8, Line 185).

**Response to Reviewer 1:**

Thank you for addressing my comments. The manuscript has improved significantly.
The only issue that was not corrected is the lack of adjustment for confounders. I was hoping the authors would use regression analysis to control for confounders such as socioeconomic status (parental income or education), family structure (2 parents vs. single parent, number of siblings), anxiety or depression, in addition to age and sex.
Are these variables available in the survey? If one or more variable is available, I recommend using regression analysis to control for measured confounders.

Thank you for your feedback, and we are pleased to hear that you found the manuscript improvements significant. Regarding your concern about the lack of adjustment for confounders, we appreciate your suggestion to utilize regression analysis to control for additional variables such as socioeconomic status, family structure, and mental health factors. Unfortunately, the survey data only includes information on age and gender, limiting our ability to adjust for a broader range of confounders. However, we have taken your recommendation into consideration and conducted additional analyses to adjust for age and gender in our revised manuscript. As a result of these adjustments, we made minor modifications to Table 4 (P12, Line 237).

Thank you for your valuable suggestions. We are looking forward to hearing from you and receiving your comments.

Best regards

**Response to Reviewer 2:**

Thank you for your response. I hope to see more research done later about this matter.

Thank you for your continued interest and thoughtful consideration of our manuscript. We appreciate your encouragement for further research on this matter.

We are looking forward to hearing from you and receiving your comments.

Best regards
